# Cardiovascular risk factors: The effects of ageing and smoking on the immune system, an observational clinical study

**DOI:** 10.3389/fimmu.2022.968815

**Published:** 2022-09-15

**Authors:** H. W. Grievink, V. Smit, B. W. Huisman, P. Gal, Y. Yavuz, C. Klerks, C. J. Binder, I. Bot, J. Kuiper, A. C. Foks, M. Moerland

**Affiliations:** ^1^ Centre for Human Drug Research, Leiden, Netherlands; ^2^ Division of BioTherapeutics, Leiden Academic Center for Drug Research, Leiden University, Leiden, Netherlands; ^3^ Department of Gynecology and Obstetrics, Leiden University Medical Center, Leiden, Netherlands; ^4^ Department of Clinical Pharmacy and Toxicology, Leiden University Medical Center, Leiden, Netherlands; ^5^ Department of Laboratory Medicine, Medical University of Vienna, Vienna, Austria

**Keywords:** cardiovascular disease, atherosclerosis, ageing, smoking, immunomodulation

## Abstract

Currently immunomodulatory compounds are under investigation for use in patients with cardiovascular disease, caused by atherosclerosis. These trials, using recurrent cardiovascular events as endpoint, require enrollment of large patient groups. We investigated the effect of key risk factors for atherosclerosis development, ageing and smoking, on the immune system, with the objective to identify biomarkers differentiating between human populations, and potentially serving as endpoints for future phase 1B trials with immunomodulatory compounds. Blood was collected from young healthy volunteers (aged 18-25 years, n=30), young smokers (18-25 years, n=20), elderly healthy volunteers (>60 years, n=20), heavy smokers (>45 years, 15 packyears, n=11) and patients with stable coronary artery disease (CAD) (>60 years, n=27). Circulating immune cell subsets were characterized by flow cytometry, and collected plasma was evaluated by proteomics (Olink). Clear ageing effects were observed, mostly illustrated by a lower level in CD8^+^ and naïve CD4^+^ and CD8^+^ T cells, with an increase in CD4^+^ and CD8^+^ effector memory T cells in elderly healthy volunteers compared to young healthy volunteers. Heavy smokers showed a more inflammatory cellular phenotype, especially a shift in Th1/Th2 ratio: higher Th1 and lower Th2 percentages compared to young healthy volunteers. A significant decrease in circulating atheroprotective oxLDL-specific IgM was found in patients with CAD compared to young healthy volunteers. Elevated pro-inflammatory and chemotactic proteins TREM1 and CCL11 were observed in elderly volunteers compared to young volunteers. In addition, heavy smokers had an increase in pro-inflammatory cytokine IL-6 and lysosomal protein LAMP3. These data show that ageing and smoking are associated with an inflammatory immunophenotype, and that heavy smokers or aged individuals may serve as potential populations for future clinical trials investigating immunomodulatory drugs targeted for cardiovascular disease.

## Introduction

The main underlying cause of cardiovascular disease (CVD) is atherosclerosis. Atherosclerosis development starts with formation of oxidized low-density lipoprotein (oxLDL). OxLDL is taken up by macrophages which differentiate into foam cells in the vessel wall, leading to inflammation. During this process other immune cells are attracted to the area and eventually an atherosclerotic plaque is formed (1). The role of the immune system in this process is complex. A vast number of immune cell subsets play an atherogenic role, such as macrophages (2), Th1 cells (3) and B2 cells (4). Other immune cell populations are thought to be atheroprotective, generally by suppressing the immune system. Examples are regulatory T cells *via* the production of anti-inflammatory cytokines such as IL-10 (5), but also B1 cells that produce the anti-atherogenic oxLDL-specific IgM (6). Binding of oxLDL-specific IgM to oxLDL leads to a decreased uptake by macrophages and an enhanced clearance by the liver (7). The balance between pro- and anti-atherogenic cells and cytokines released by these cells is lost during atherosclerosis development.

Currently, patients that are diagnosed with CVD caused by atherosclerosis are generally treated with cholesterol-lowering drugs, beta blockers and/or anti-coagulants. While this is a successful treatment leading to a reduction in circulating LDL cholesterol, a large proportion of CVD patients remain at high risk for recurring cardiovascular events. Therefore, immunomodulation has recently gained interest as a potential therapy for atherosclerosis. In the CANTOS trial treatment with the anti-IL-1β antibody canakinumab results in fewer cardiovascular events in patients with high plasma CRP levels and previous myocardial infarction, compared to placebo (8). Furthermore, a study investigating the effect of low-dose colchicine (LoDoCo trial) on recurrent cardiovascular events, showed that colchicine treatment significantly reduces the risk of cardiovascular events (9). However, treatment of patients with low-dose methotrexate (CIRT trial) did not show an effect on myocardial infarction, stroke or cardiovascular death (10). Between these studies however, inclusion criteria differed, as patients in the CIRT and LoDoCo trials had either type 2 diabetes or metabolic syndrome in addition to previous myocardial infarction, while patients in the CANTOS trial had enhanced hsCRP levels in addition to previous myocardial infarction.

The aforementioned trials required large study groups and a long follow-up time to assess the functionality of the investigated immunomodulatory drug. Early phase clinical trials for an initial assessment of the safety, pharmacokinetics and pharmacodynamics of novel therapeutic drug candidates are however commonly conducted in relatively small groups of healthy volunteers. Evaluation of drug activity in healthy volunteers can be challenging, especially for immunomodulatory drugs, as generally, immune activation is lacking in healthy subjects. For such drugs, early single or multiple ascending dose programs in healthy volunteers are commonly enriched with *ex vivo* cell stimulation assays or *in vivo* immune challenges to evaluate drug effects. Alternatively, early inclusion of a small group of subjects with differentiating immune endpoints or patients can be considered: in a phase 1B setting, the effect of one or a few drug doses on specific immune endpoints could provide insight into the dose-activity relationship.

Because of the immunological basis underlying the pathophysiology of atherosclerosis, and the potential value of well-characterized populations with an altered immune system/response for early phase clinical pharmacology trials, we characterized immune system parameters in small groups of volunteers, stratified for age, smoking behavior, and disease. We investigated the effect of ageing and smoking, known risk factors for atherosclerosis development, on circulating immune cell phenotype, immune cell functionality, and circulating inflammatory protein levels. We included young healthy volunteers (aged 18 – 25 years), elderly healthy volunteers (>60 years), young smokers (18 – 25 years), and heavy smokers (>45 years) in the study, and also enrolled stable coronary artery disease (CAD) patients (>60 years) as reference population. We aimed to identify immune endpoints that clearly differentiated between volunteer groups, so that ultimately these populations could serve as ‘disease model’ to run small phase 1B studies with immunomodulators under development for cardiovascular disease, thereby circumventing the use of patients with CAD in these trials due to possibly interfering medication use by these patients.

## Materials and methods

### Subjects

**Figure 1:**
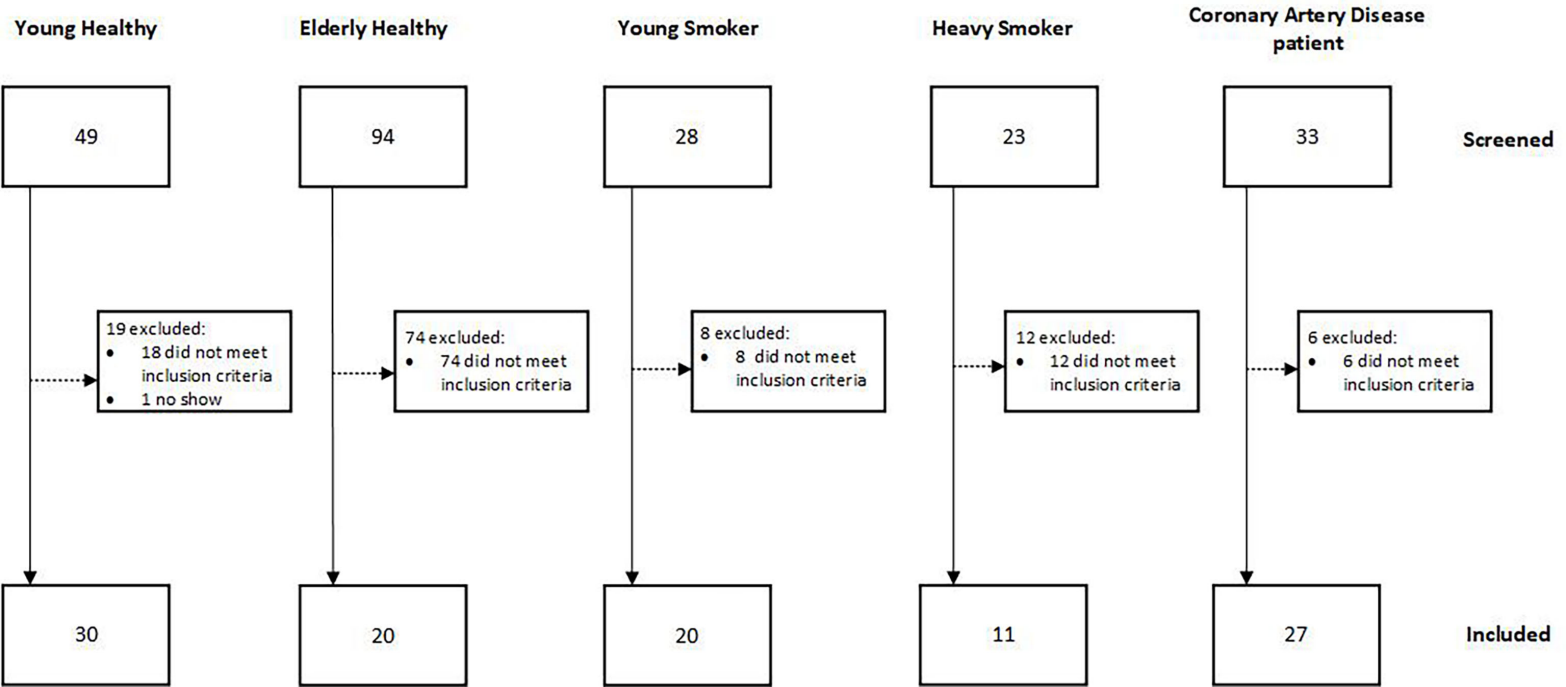
CONSORT flowchart.

In total 108 male subjects were enrolled between April 2019 and March 2020 (**Figure 1**). The study took place at the Centre of Human Drug Research in Leiden, the Netherlands. The study was approved by the Independent Ethics Committee of the Foundation “Evaluation of Ethics in Biomedical Research” (Stichting Beoordeling Ethiek Biomedisch Onderzoek), Assen, the Netherlands and Declaration of Helsinki principles were followed. The study is registered in the Dutch Trial Register (Nederlands Trial Register (NTR)) under number NL7754. All subjects signed an informed consent form prior to any study‐related activity.

Five groups of subjects were included: young healthy volunteers (YH) aged between 18 and 25, elderly healthy volunteers (EH) aged >60 years, young smokers (YS) aged 18-25 years, smoking 8 cigarettes/day for at least 6 months, heavy smokers (HS) aged >45 years smoking at least 15 pack years and stable coronary artery disease patients (CAD) aged >60 years. The CAD group was considered stable after undergoing a revascularization procedure and being without recurrent events for at least 1 year. Subjects were not allowed to take any medication, except for the CAD group. Subjects underwent a general medical screening prior to inclusion in the study and standard chemistry and hematology tests were performed at clinical chemistry lab of the Leiden University Medical Center. BMI was restricted between 18 and 28 inclusive. Subjects for the YH, EH and YS groups were excluded if the following risk factors for CAD were present: high cholesterol, smoking, diabetes, hypertension or familial risk. Subjects for the YH and EH groups were considered healthy when no abnormalities were found in the urinalysis, hematology and chemistry lab tests, including liver enzymes, kidney function markers, cardiovascular markers (cholesterol, triglycerides, lipoprotein A, NTproBNP), CRP and coagulation markers. Furthermore, no abnormalities in the physical examination, including blood pressure and electrocardiogram and in the medical history were found.

### PBMC isolation and cryopreservation

PBMCs were isolated using Cell Preparation Tubes (Becton Dickinson, Frankin Lakes, NJ, USA), according to manufacturer’s protocol. In short, tubes are centrifuged at 1800xg for 30 minutes after which the PBMCs were removed. PBMCs were washed twice using PBS (pH 7.2, Gibco, Thermo Fisher Scientific, Waltham, MA, USA). PBMCs were dissolved in heat inactivated fetal bovine serum (Gibco, Thermo Fisher Scientific), containing 10% DMSO (Sigma-Aldrich, Deisenhofen, Germany). PBMCs were frozen using a CoolCell at -80°C, prior to storage in liquid nitrogen.

### B cell isolation and stimulation

B cells were isolated directly from whole blood with the Easysep Direct B cell isolation kit using the RoboSep (Stemcell, Vancouver, Canada). Purity was assessed by flow cytometry using CD45-PE (clone: HI30) and CD19-APC (clone: HIB19) antibodies, and was >90% for all samples. B cells were stimulated with CpG class B (ODN2006, *In vivo*gen, Toulouse, France) or PBS for 24 hours. After 24 hours expression of activation markers CD69, CD80 and CD86 were measured by flow cytometry, using CD69-AF700 (clone: FN50), CD80-PE (clone: 2D10) and CD86-APC (clone: BU63). All antibodies were from Biolegend (San Diego, CA, USA).

### Whole blood stimulation and cytokine measurement

Heparinized whole blood was stimulated with 2ng/ml lipopolysaccharide (LPS O111:B4, Sigma-Aldrich) for 6 hours or PMA and ionomycin (both Sigma-Aldrich) + Brefeldin A (Thermo Fisher) for 4 hours at 37°C and 5% CO2. PMA and ionomycin stimulated samples were used for intracellular cytokine staining. LPS stimulated samples were centrifuged and supernatant was collected. Cytokines in supernatant were measured using the LegendPlex virus response panel (Biolegend) according to manufacturer’s manual.

### Flow cytometry

Red blood cell lysis was performed on whole blood samples using RBC lysis buffer (Thermo Fisher Scientific). Leukocytes were stained with fluorochrome labeled antibodies as indicated in **Supplementary Table I**. Intracellular staining was performed after fixation and permeabilization with IC fixation buffer and permeabilization buffer (both Thermo Fisher Scientific). Samples were measured on a MacsQuant 16 analyzer (Miltenyi Biotec, Bergisch-Gladbach, Germany) and analyzed using Flowlogic software (Inivai, Mentone, Australia).

### Immunoglobulin ELISAs

K_2_EDTA plasma antibody levels to PC-BSA and CuSO4-oxidized LDL (oxLDL) were measured by chemiluminescent ELISA as reported previously (11). In brief, PC-BSA (Biosearch Technologies) and oxLDL were coated at 5µg/ml in PBS/EDTA. IgM antibodies were measured at a dilution of 1:500 and IgG antibodies at 1:1000.

### oxLDL IgM B cell ELISpot

IgM B cell ELISpots were performed on thawed PBMCs, using the IgM ELISpot Basic kit (Mabtech, Stockholm, Sweden). PBMCs were stimulated with R848 and IL-2 for 3 days to stimulate antibody production. ELISpot plates (Multiscreen IP filter plate, PVDF membrane, Sigma-Aldrich) were coated with 15µg/ml oxLDL (Thermo Fisher Scientific) or anti-IgM antibody. PBMCs were incubated in the coated ELISpot plates at 200.000 (oxLDL) or 12.500 cells/well (anti-IgM) for 24 hours in X-Vivo15 medium (Lonza, Ambiose, France) with 1% penicillin and streptomycin (Thermo Fisher Scientific). Plates were developed using detection antibodies from the kit and developed using NCIB/NBT substrate (Mabtech). Spots were counted using the Bioreader 6000-E β (Biosys, Karben, Germany). Samples were tested in triplicate. Mean oxLDL spots/total IgM spot count were calculated.

### Proteomics

92 proteins were measured in multiplex in K_2_EDTA plasma by Olink (Uppsala, Sweden) using the pre-designed immune response panel. Protein levels were measured using oligonucleotide labeled antibodies. When 2 antibodies were in proximity, the DNA sequence was enhanced by real time PCR. The results were expressed on a log2 scale as normalized protein expression (NPX).

### Statistical analysis

The means of all groups were compared to each other, and pairwise differences were calculated using one-way ANOVA (with Dunnett’s multiple testing adjustment) or Kruskal-Wallis test (Dunn’s multiple testing adjustment) when normality assumption failed. P values ≤0.05 obtained from above tests were considered statistically significant. Data are expressed as arithmetic mean ± standard deviation. Analysis and visualization were done using Graphpad Prism version 9.2.0 (Graphpad Sofware, San Diego, CA, USA).

Proteomics data were analyzed using one-way ANOVA (with Tukey’s multiple testing adjustment for multiple groups) and corrected for multiple testing using Benjamini-Hochberg procedure using SPSS (IBM, Armonk, NY, USA). Hierarchical cluster analysis was performed using agglomeration method of Ward’s minimum variance on Euclidian dissimilarities matrix based on data in original scale. Hierarchical cluster analysis was conducted using functions {R package} in Rstudio (Boston, MA, USA) for clustering and visualization of its results in forms of a heatmap are hclust {stat} and pheatmap {pheatmap} respectively.

## Results

In total, 108 subjects were included in the study as shown in **Figure 1** and **Table 1** (subject demographics). The main exclusion factor for elderly healthy volunteers was an increased cholesterol level (reference range <5.00 mmol cholesterol/L and <3.00 mmol LDL cholesterol/L) or hypertension (systolic >140 mmHg and/or diastolic >90 mmHg in supine position after 5 minutes rest). Twelve of the 27 coronary artery disease patients were smokers. 26 of 27 were taking medication related to CVD, 24/27 used NSAIDs, 21/27 used statins, 8/27 used beta blockers, 4/27 used calcium blockers, 4/27 used angiotensin II blockers and 2/27 used diuretics.

**Table 1:** Demographics.

Group	Young Healthy(n = 30)	Elderly Healthy(n = 20)	Young Smoker(n = 20)	Heavy Smoker(n = 11)	Coronary Artery Disease patients (n = 27)	P value
age years median (Q25 - Q75)	22 (19 - 22.3)	70.5^a,b,c^ (67 - 77.5)	22 (21 - 23.8)	55^a,b^ (48 - 68)	69.5^a,b,c^ (66 - 73.3)	<0.0001
BMI kg/m^2^ median (Q25 - Q75)	22.5 (20.6 - 23.3)	24.6 ^a,b^ (22.8 - 25.4)	21.7 (20.7 - 23.8)	25.2 ^a,b^ (22.8 - 27.2)	24.8 ^a,b^ (23.5 - 26.4)	<0.0001
diastolic bp (supine) mm Hg mean ± SD	69.9 ± 4.8	81.4 ± 8.9 a^,b^	72.4 ± 6.7	83.1 ± 5.5 a^,b^	80.7 ± 9.3 a^,b^	<0.0001
systolic bp (supine) mm Hg mean ± SD	118 ± 9.2	133.4 ± 15^a^	122 ± 10.3	127.6 ± 11.4	131.8 ± 17.6^a^	0.0002
cholesterol mmol/L mean ± SD	3.84 ± 0.54	4.53 ± 0.43^a,b^	3.88 ± 0.57	5.19 ± 0.97^a,b^	4.22 ± 0.98^c^	<0.0001
LDL mmol/L mean ± SD	2.19 ± 0.36	2.60 ± 0.37^b^	2.10 ± 0.37	3.17 ± 0.95^a,b^	2.30 ± 0.93^c^	0.0002
HDL mmol/L mean ± SD	1.29 ± 0.21	1.46 ± 0.28	1.40 ± 0.32	1.32 ± 0.28	1.50 ± 0.33	0.1249
triglycerides mmol/L mean ± SD	0.78 ± 0.32	1.04 ± 0.26^c^	0.83 ± 0.30	1.55 ± 1.06^a,b^	1.03 ± 0.50^c^	0.0003
Apolipoprotein A, g/L mean ± SD	1.32 ± 0.17	1.44 ± 0.21	1.33 ± 0.21	1.37 ± 0.22	1.49 ± 0.25^a^	0.03
NTproBNP, Mean	26.52 ± 17.16	94.38 ± 87.96^a,b^	29.40 ± 22.59	48.96 ± 43.53	175.5 ± 182.6^a,b,c^	<0.0001

^a^ = significantly different to YH. ^b^ = significantly different to YS, ^c^ = significantly different to HS.

### Elevated total leukocyte and neutrophil numbers in aged volunteers, additionally enhanced by smoking

**Figure 2:**
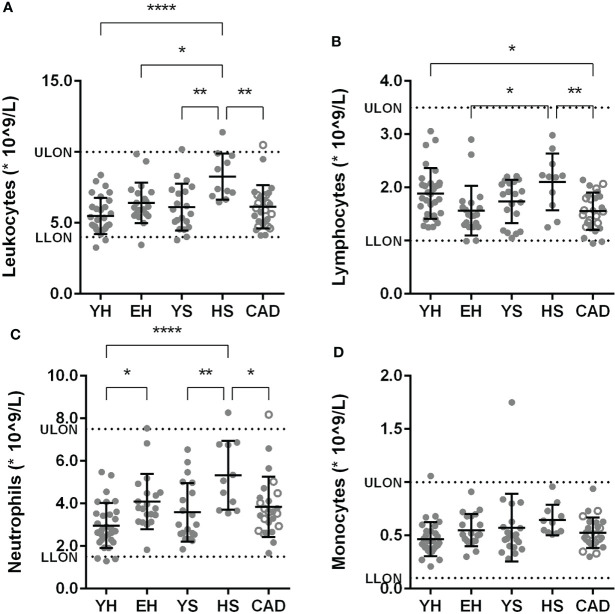
Effect of ageing and smoking on absolute circulating leukocytes. Amount of leukocytes **(A)**, lymphocytes **(B)**, neutrophils **(C)**, monocytes **(D)** in circulation as measured by sysmex. Mean ± SD are shown. LLON, lower limit of normal; ULON, upper limit of normal; YH, young healthy; EH, elderly healthy; YS, young smoker; HS, heavy smoker; CAD, coronary artery disease patient. Open circles in the CAD group represent smokers. Statistics was performed using one-way ANOVA with Dunnett’s *post hoc* test, means were compared to the YH group. P values ≤0.05 are considered significant. * p ≤ 0.05, ** p ≤ 0.005, **** p≤ 0.0001.

First, we compared the absolute numbers of circulating leukocytes and its subsets between the groups. In heavy smokers, the total number of leukocytes was significantly higher compared to all other groups (8.3 × 10^9^/L ± 1.6 for HS, vs 5.4 ± 1.3 × 10^9^/L for YH, 6.4 ± 1.4 × 10^9^/L for EH, 6.1 ± 1.6 × 10^9^/L for YS, 6.1 ± 1.5 × 10^9^/L for CAD) (**Figure 2A**). The number of lymphocytes was lower in the CAD group compared to young healthy volunteers and heavy smokers (1.6 ± 0.3 × 10^9^/L for CAD, vs 1.89 ± 0.47 × 10^9^/L for YH and 2.1 ± 0.5 × 10^9^/L for HS), while the numbers were significantly lower in the elderly healthy group (1.6 ± 0.5 × 10^9^/L) compared to the heavy smokers (**Figure 2B**). The number of neutrophils was significantly higher in elderly healthy subjects compared to young healthy subjects (4.1 ± 1.3 × 10^9^/L for EH, vs 3.0 ± 1.1 × 10^9^/L for YH) (**Figure 2C**), and even higher in the heavy smoker group (5.3 ± 1.6 × 10^9^/L). Furthermore, neutrophil numbers were significantly higher in the heavy smokers compared to the CAD group (3.8 ± 1.4 × 10^9^/L). No differences were observed in numbers of circulating monocytes (**Figure 2D**).

### Lower levels of CD16^+^ monocytes and plasmacytoid dendritic cells in smoking volunteers

**Figure 3:**
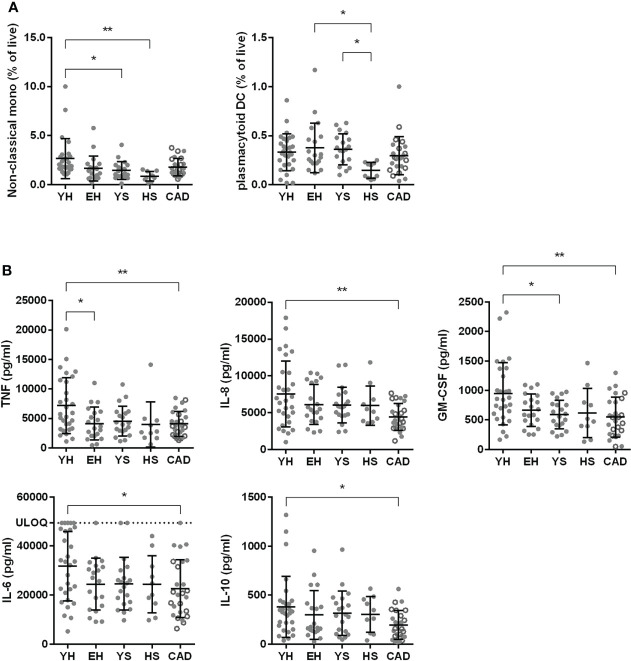
Effect of ageing and smoking on myeloid cells. Percentage of non-classical monocytes (CD14^-^ CD16^+^) and plasmacytoid dendritic cells (HLA-DR^+^, CD19^-^, CD14^-^, CD123^+^) in whole blood as assessed by flow cytometry **(A)**. Whole blood was stimulated with 2 ng/ml LPS and cytokine release was measured using LegendPlex **(B)**. YH, young healthy; EH, elderly healthy; YS, young smoker; HS, heavy smoker; CAD, coronary artery disease patient. Open circles in the CAD group represent smokers. Statistics was performed using one-way ANOVA with Dunnett’s *post hoc* test, means were compared to the YH group. P values ≤0.05 are considered significant. * p ≤ 0.05, ** p ≤ 0.005.

Upon flow cytometry analysis of the myeloid cells, a lower percentage of non-classical (CD14^-^CD16^+^) monocytes was seen in the heavy smoker and young smoker groups, compared to young healthy volunteers (0.9 ± 0.5% for HS, 1.5 ± 0.9% for YS compared to 2.7 ± 2.0% for YH). Classical (CD14^+^CD16^-^) and intermediate (CD14^+^CD16^+^) monocytes did not differ between the groups (**Figure S1A**). The percentage of plasmacytoid DCs (HLA-DR^+^ CD14^-^ CD123^+^) was significantly lower in heavy smokers compared to elderly healthy volunteers and young smokers (0.15 ± 0.08% for HS vs. 0.36 ± 0.16% for YS and 0.33 ± 0.19% for YH, **Figure 3A**).

To investigate the functionality of (mainly) myeloid cells, whole blood was stimulated with TLR4 ligand LPS, after which cytokine release was measured in the supernatant. A significant decrease in TNFα release was found in elderly healthy volunteers and patients with CAD compared to young healthy (**Figure 3B**, see **Table S2** for mean and SD). GM-CSF was significantly decreased in the young smokers and CAD patient groups compared to young healthy volunteers. The interleukins IL-8, IL-6 and IL-10 were significantly decreased in the CAD group compared to young healthy volunteers. No differences were observed in IL-1β release (**Figure S1B**).

### Reduced naïve T cell levels in elderly healthy volunteers, while smokers have an elevated relative pro-inflammatory Th1 fraction

**Figure 4:**
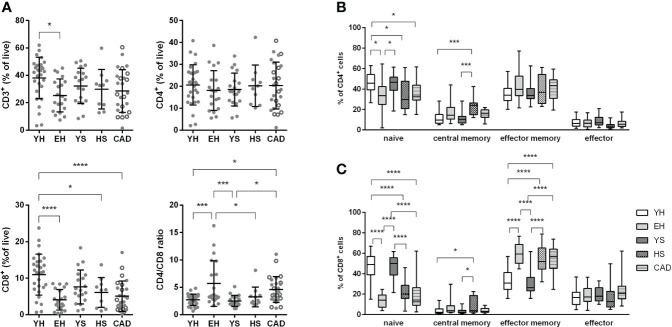
Effect of ageing and smoking on T cells and memory subsets. Percentage of CD3^+^, CD4^+^ and CD8^+^ T cells of total CD45^+^ leukocytes was measured by flow cytometry **(A)**. Naïve (CCR7^+^ CD45ro^-^) and memory subsets (central memory: CCR7^+^ CD45ro^+^, effector memory: CCR7^-^ CD45ro^+^) were quantified for CD4^+^ T cells **(B)** as well as CD8^+^ T cells **(C)**. YH, young healthy; EH, elderly healthy; YS, young smoker; HS, heavy smoker; CAD, coronary artery disease patient. Open circles in the CAD group represent smokers. Statistics was performed using one-way ANOVA with Dunnett’s *post hoc* test, means were compared to the YH group. P values ≤0.05 are considered significant. * p ≤ 0.05, *** p ≤ 0.001, **** p≤ 0.0001.

Circulating levels of T cell subsets were assessed using flow cytometry. CD3^+^ T cells as percentage of CD45 were lower in aged volunteers (25.3 ± 12.1% for EH, vs 38.1 ± 15.1% for YH) (**Figure 4A**). This difference can be attributed to a strong decrease in CD8^+^ cytotoxic T cells (4.1 ± 2.8% for EH, vs 11 ± 5.6% for YH), which was also observed in the heavy smoker and CAD patient groups (6.1 ± 4.1% for HS, 5.1 ± 4.1% for CAD). Furthermore, the CD4/CD8 ratio, calculated using percentages of CD3+ cells, was increased in the elderly healthy and CAD patient groups (5.7 ± 4.1 for EH, 4.6 ± 2.4 for CAD vs. 2.7 ± 1.0 for YH, 2.5 ± 1.0 for YS and 3.3 ± 1.8 for HS). As expected, the relative number of naive cells (CCR7^+^ CD45ro^-^) in both the CD4^+^ and CD8^+^ T cell populations decreases significantly with age, while the percentage of memory cells increases (**Figures 4B, C**). Interestingly, both CD4^+^ and CD8^+^ central memory (CCR7^+^ CD45ro^+^) cell levels were elevated in heavy smokers compared to young volunteers (22.3 ± 8.9% for HS vs. 12.1 ± 6.8% for YH and 10.8 ± 5.8% for YS, CD8: 8.6 ± 7.9% for HS vs. 3.7 ± 3.4% for YH and 3.3 ± 2.7% for YS) (**Figures 4B, C**).

**Figure 5:**
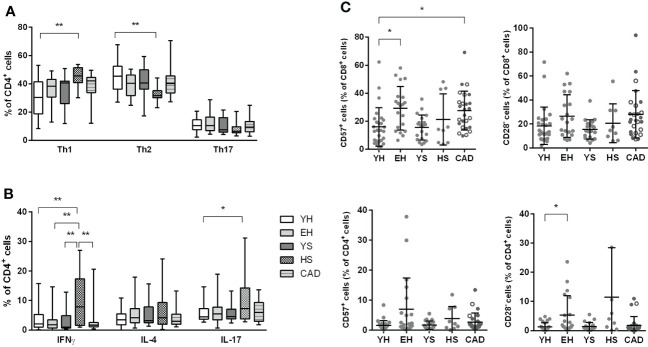
Effect of ageing and smoking on Th subsets and T cell senescence. Percentage of Th1 (CXCR3^+^ CCR6^-^), Th2 (CXCR3^-^ CCR6^-^) and Th17 (CXCR3^-^ CCR6^+^) cells were measured by flow cytometry **(A)**. Cytokine production in CD4^+^ T cells was measured after stimulation with PMA + ionomycin in the presence of BrefA, using flow cytometry **(B)**. Both CD4^+^ as well as CD8^+^ T cells were assessed for senescence markers CD57 (positive) and CD28 (negative) **(C)**. YH = young healthy, EH = elderly healthy, YS, young smoker; HS, heavy smoker; CAD, coronary artery disease patient. Open circles in the CAD group represent smokers. Statistics was performed using one-way ANOVA with Dunnett’s *post hoc* test, means were compared to the YH group. P values ≤0.05 are considered significant. * p ≤ 0.05, ** p ≤ 0.005.

Upon focusing on the Th subsets, we observed that heavy smokers had a larger Th1 (CXCR3^+^ CCR6^-^) fraction (45.4 ± 7.1% for HS vs 30.8 ± 12.8% for YH), and a smaller Th2 (CXCR3^-^ CCR6^-^) fraction of CD4^+^ T cells (32.7 ± 5.9% for HS vs 45.4 ± 11.4% for YH) (**Figure 5A**). No differences were observed in the regulatory T cell subset (**Figure S2**).

To evaluate the functionality of CD4^+^ T cells in the different populations, whole blood was stimulated with PMA and ionomycin for 4 hours in the presence of brefeldin A for intracellular cytokine staining in T cells (**Figure 5B**). Corresponding to the elevated Th1 cells, more IFNγ producing CD4^+^ cells were observed in heavy smokers compared to all other groups (9.7 ± 9.1% for HS vs. 3.7 ± 3.8% for YH, 2.7 ± 3.4% for EH, 3.4 ± 4.0% for YS and 2.9 ± 4.1% for CAD). Although Th17 (CXCR3^-^ CCR6^+^) cell percentages were unaltered in heavy smokers (**Figure 5A**), we observed an elevated percentage of IL-17 producing CD4^+^ T cells in this group, compared to young healthy volunteers (10.2 ± 9.2% for HS vs. 5.7 ± 3.0% for YH).

Accumulation of CD57^+^ and CD28^null^ T cells in the blood has been described upon ageing (12, 13). Indeed, we found increased CD57^+^ cells within the CD8^+^ T cells of elderly healthy and patients with CAD, compared to young healthy volunteers (29.3 ± 15.5% for EH; 27.5 ± 13.9% for CAD vs. 16.0 ± 13.8% for YH) (**Figure 5C**). Similarly, we observed a trend (p=0.06) towards increased CD28^null^ cells in the CD8^+^ T cell population in patients with CAD (28.0 ± 19.7%) compared to young healthy volunteers (18.5 ± 15.6%). Although we did not observe any significant differences in CD57^+^ cells within the CD4^+^ T cell compartment, we found elevated levels of CD28^null^ cells in CD4^+^ T cells of elderly healthy compared to young healthy volunteers (5.3 ± 6.7% for EH vs. 1.3 ± 1.4% for YH). The percentage of CD57^+^ and CD28^null^ CD4^+^ T cells in patients with CAD remained unaltered.

### Patients with CAD have lower levels of circulating IgM

**Figure 6:**
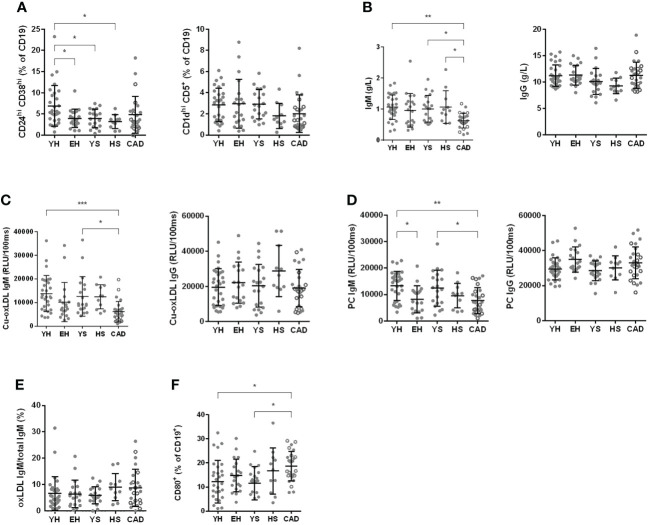
Effect of ageing and smoking on B cells. Number of CD24^hi^ CD38^hi^ and CD1d^hi^ CD5^+^ regulatory B cells, evaluated by flow cytometry **(A)**. Total IgM and IgG as assessed at the clinical chemistry lab at LUMC **(B)**. oxLDL specific IgM and IgG **(C)** as well as PC IgM and IgG **(D)** as assessed by ELISA. oxLDL secreting B cells were measured using ELISpot after 3 days of stimulation with R848 and IL-2 and are expressed as percentage of total IgM secreting B cells **(E)**. CD80 expression was measured by flow cytometry on isolated B cells after 24 hours of stimulation with CpG B **(F)**. YH, young healthy; EH, elderly healthy; YS, young smoker; HS, heavy smoker; CAD, coronary artery disease patient. Open circles in the CAD group represent the smokers. Statistics was performed using one-way ANOVA with Dunnett’s *post hoc* test or Kruskall-Wallis test with Dunn’s *post hoc* test, means were compared to the YH group. P values ≤0.05 are considered significant. * p ≤ 0.05, ** p ≤ 0.005, *** p ≤ 0.001.

Next, we investigated humoral immunity, starting with circulating B cell subsets. No differences were observed in naive, CD19^+^ CD27^+^ CD43^+^ B1 cells, non-class-switched or class-switched B cells for any group compared to the healthy young volunteers (**Figure S3**). The percentage of transitional B cells was significantly lower in aged volunteers (4.0 ± 2.2% for EH), young smokers (3.9 ± 2.2% for YS) and heavy smokers (3.2 ± 1.7% for HS) compared to young healthy volunteers (6.9 ± 4.8%). No significant differences were found for CD5^+^ CD1d^hi^ regulatory B cells (**Figure 6A**).

Next, we measured circulating immunoglobulin levels. We observed a significantly lower concentration of total serum IgM in the CAD patient group (0.63 ± 0.24 g/L) compared to young healthy volunteers (1.07 ± 0.40 g/L), young smokers (1.01 ± 0.43 g/L) and heavy smokers (1.07 ± 0.53 g/L) (**Figure 6B**). No differences were found for total IgG serum concentrations (**Figure 6B**). We also measured oxLDL-specific immunoglobulins, as these immunoglobulins can play both anti- (IgM) as well as pro-atherogenic (IgG) roles. The level of oxLDL-specific IgM was significantly lower in the CAD patient group compared to both young healthy volunteers and young smokers (6146 ± 4336 RLU for CAD vs. 13828 ± 7620 RLU for YH and 12636 ± 8349 RLU for YS), while no differences were observed in oxLDL-specific IgG values (**Figure 6C**). Since phosphorylcholine (PC) is a specific epitope of oxLDL, PC-specific antibodies were measured as well. In elderly healthy volunteers, a lower level of PC-specific IgM was found (8176 ± 5114 RLU for EH vs. 13281 ± 5512 RLU for YH), while the PC-specific IgG signal was elevated (34925 ± 7138 RLU for EH vs. 29502 ± 6281 RLU for YH) (**Figure 6D**). The lowered PC-IgM was also observed in patients with CAD (7696 ± 4908 RLU).

To assess the number of oxLDL-IgM secreting B cells present in PBMCs, PBMCs were stimulated with R848 (TLR7/8 agonist) and IL-2 for 2 days to activate memory B cells, after which the number of IgM and oxLDL-specific IgM secreting B cells was measured using ELISpot. The percentage of oxLDL-specific IgM secreting B cells of total IgM secreting B cells is shown in **Figure 6E**. Although the circulating levels of oxLDL-IgM are decreased in the patients with CAD, the number of oxLDL-specific IgM secreting B cells is not.

To assess the activation capacity of B cells, B cells were stimulated with CpG class B for 24 hours, and activation was measured by CD69, CD80 and CD86 expression using flow cytometry. No differences were observed between groups for the expression of CD69 and CD86 (**Figure S3**). Expression of the co-stimulation marker CD80 was significantly increased on B cells in the CAD patient group, compared to young healthy volunteers and young smokers (18.59 ± 6.15% for CAD vs. 12.16 ± 8.88% for YH and 11.59 ± 6.91% for YS) (**Figure 6F**).

### Elevated TREM1 and CCL11 plasma levels in elderly (smoking) subjects and patients with CAD

**Figure 7:**
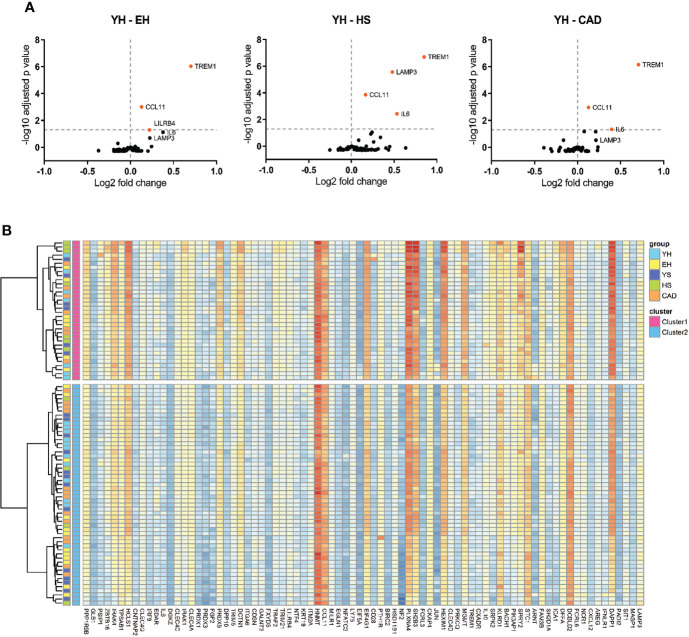
Effect of ageing and smoking on levels of circulating inflammatory proteins. Volcano plots of proteomics **(A)**, EH vs YH, HS vs YH and CAD vs YH. Horizontal line depicts significance, data points depicted in orange show increased serum expression. Heatmap of agglomerative hierarchical clustering using Ward’s method of proteomics data **(B)**. YH (n=19), EH (n=19), YS (n=19), HS (n=11), CAD (n=20). YH, young healthy; EH, elderly healthy; YS, young smoker; HS, heavy smoker; CAD, coronary artery disease patient.

Plasma samples were analyzed for inflammatory proteins using the Olink immune response panel (**Figure 7**). A significantly elevated level of triggering receptor expressed on myeloid cells 1 (TREM1), C-C motif chemokine ligand 11 (CCL11) and leukocyte immunoglobulin like receptor B4 (LILRB4) was seen in plasma of elderly healthy volunteers compared to young healthy volunteers. In heavy smokers the elevation of TREM1 and CCL11 was more profound, and also significantly elevated levels of IL-6 and lysosomal associated membrane protein 3 (LAMP3) were found. Patients with CAD show a similar profile to elderly healthy volunteers. When clustering all data using Ward’s hierarchical clustering, two clusters were found, consisting of samples from all subject groups (**Figure 7B**). This shows that the protein profiles do not clearly differentiate the groups from each other.

## Discussion

Immunomodulation has shown to be a valuable therapeutic approach in atherosclerosis in animal studies (14). Recently, clinical trials with immunomodulatory drugs have been performed in cardiovascular patient populations. In the CANTOS and LoDoCo trials (8, 9) a significant reduction in cardiovascular risk was observed, while low dose methotrexate (CIRT) did not reveal a therapeutic effect (10). Since using recurrent cardiovascular events as endpoint requires large patient groups, clinical evaluation of immunomodulatory investigational drugs for atherosclerosis could benefit from relatively small phase 1B studies, in well-characterized populations carrying cardiovascular risk factors (ageing, smoking). We conducted an observational study evaluating the effects of age, smoking and cardiovascular disease on a broad set of immune endpoints. We aimed to identify immune markers differentiating populations with cardiovascular risk factors from young healthy volunteers, thereby identifying the most optimal population to be used as ‘disease model’ for future phase 1B studies with immunomodulatory drugs.

First, we assessed the effects of ageing on circulating immune cell populations. A hallmark of immunosenescence is decreased lymphopoiesis and accumulation of senescent cells (15, 16). Indeed, we observed significantly reduced total lymphocyte numbers, total CD3^+^ T cells and CD8^+^ T cells in aged populations. Furthermore, we observed an increase in senescent CD4^+^CD28^null^ cells in elderly healthy volunteers and elevated CD8^+^CD57^+^ T cells in both elderly healthy volunteers and stable CAD patients, compared to young individuals. Previous studies have shown that a high frequency of senescent CD28^null^ and CD57^+^ T cells in peripheral blood and/or atherosclerotic plaques strongly associate with hyperglycemia, acute cardiovascular events and mortality (17–19). Interestingly, statin treatment has been shown to reduce elevated senescent CD4^+^CD28^null^ cells (17), which might attribute to unaltered CD4^+^CD28^null^ levels in statin treated CAD patients compared to young healthy, whereas levels in elderly healthy increased. Whether statin treatment might have reduced the frequency of CD8^+^ CD57^+^ T cells in the CAD patient group, remains to be elucidated.

Besides ageing, smoking is an important risk factor for atherosclerosis development as well (20). An increase in central memory T cells was observed in the heavy smoker group, which has been described previously, where the amount of CD45ro^+^ T cells was strongly correlated to the amount of pack years (21). In addition, an increase in the Th1 fraction was seen in this population, with a corresponding increase in IFNγ producing T cells.

The elderly healthy group resembled the CAD group quite well, with the exception of B cell effects, and myeloid cell function after LPS stimulation. A lower level of oxLDL-specific IgM was found in patients with CAD compared to young healthy volunteers, as was also shown previously (22, 23). OxLDL IgM plays an atheroprotective role, it facilitates the clearance of apoptotic cells (7, 24), while preventing foam cell formation by blocking oxLDL uptake by macrophages (25), and possesses antithrombotic properties (26). Furthermore, oxLDL IgM has an inverse relation to human intima media carotid artery thickness (27). A reduced level of PC-specific IgM was also found in the patients with CAD and elderly healthy volunteers, consistent with a decline of these type of IgM with age, which are considered atheroprotective (28, 29).

Myeloid cell functionality was evaluated by performance of whole blood LPS challenges. Overall, cytokine release was significantly reduced in the CAD group compared to young healthy volunteers. This decrease in cytokine production in the stable CAD group could be attributed to medication use. Statins are known to have an immunosuppressive effect (30), supporting its use in graft versus host disease (31), multiple sclerosis (32), and heart transplant patients (33). However, it has also been shown that statins do not affect cytokine production by monocytes after LPS stimulation (34).

Finally, we compared the study groups for circulating inflammatory markers by proteomics. A significant increase in plasma TREM1 and CCL11 was found in elderly volunteers compared to the young healthy group. TREM1 is an enhancer of pro-inflammatory innate immune responses and plays an important role during infection. Increased TREM1 levels have been found in Alzheimer’s disease (35), Parkinson’s disease (36) and sepsis (37), but to our knowledge this is the first time an increase in plasma TREM1 levels is found in ageing. CCL11 (eotaxin) plasma levels have been found to positively correlate to the score of coronary artery stenosis (38). In both heavy smokers and patients with CAD the increase in TREM1 and CCL11 was also found, indicating that an increase in these proteins may be potential risk factors for atherosclerosis development. Both groups had increased IL-6 plasma levels as well, IL-6 being one of the hallmarks of inflammaging and influenced by smoking (39, 40). Increased plasma IL-6 levels correlates with risk for CAD (41–43), furthermore treatment of patients with high atherosclerotic risk with ziltivekimab, a human monoclonal antibody against IL-6, resulted in a reduction of inflammation biomarkers relevant to atherosclerosis (44). LAMP3 clearly distinguished the heavy smoker group from the other groups. LAMP3 is mainly expressed in mature dendritic cells, mostly in the lysosomes. Little is known about the role of LAMP3 in disease, although an association between LAMP3 expression and serum auto-antibodies in Sjögren’s syndrome was found (45).

There are several limitations to this study. TLR4, through which LPS primarily signals, is not solely expressed on myeloid cells (46), so we cannot exclude the possibility that non-myeloid cells may have contributed to the observed cytokine responses. Furthermore, differences in whole blood composition between the groups could attribute to the observed differences in cytokine production between the groups. Another limitation of this study is the small sample size, and the relatively high analytical variation of several endpoints. While clear immunological differences were found between study groups, smaller differences could have remained unnoticed. However, it was the specific aim of this study to identify differentiating immune endpoints at sample sizes resembling future phase 1B studies, which are commonly conducted at relatively low volunteer numbers.

**Figure 8:**
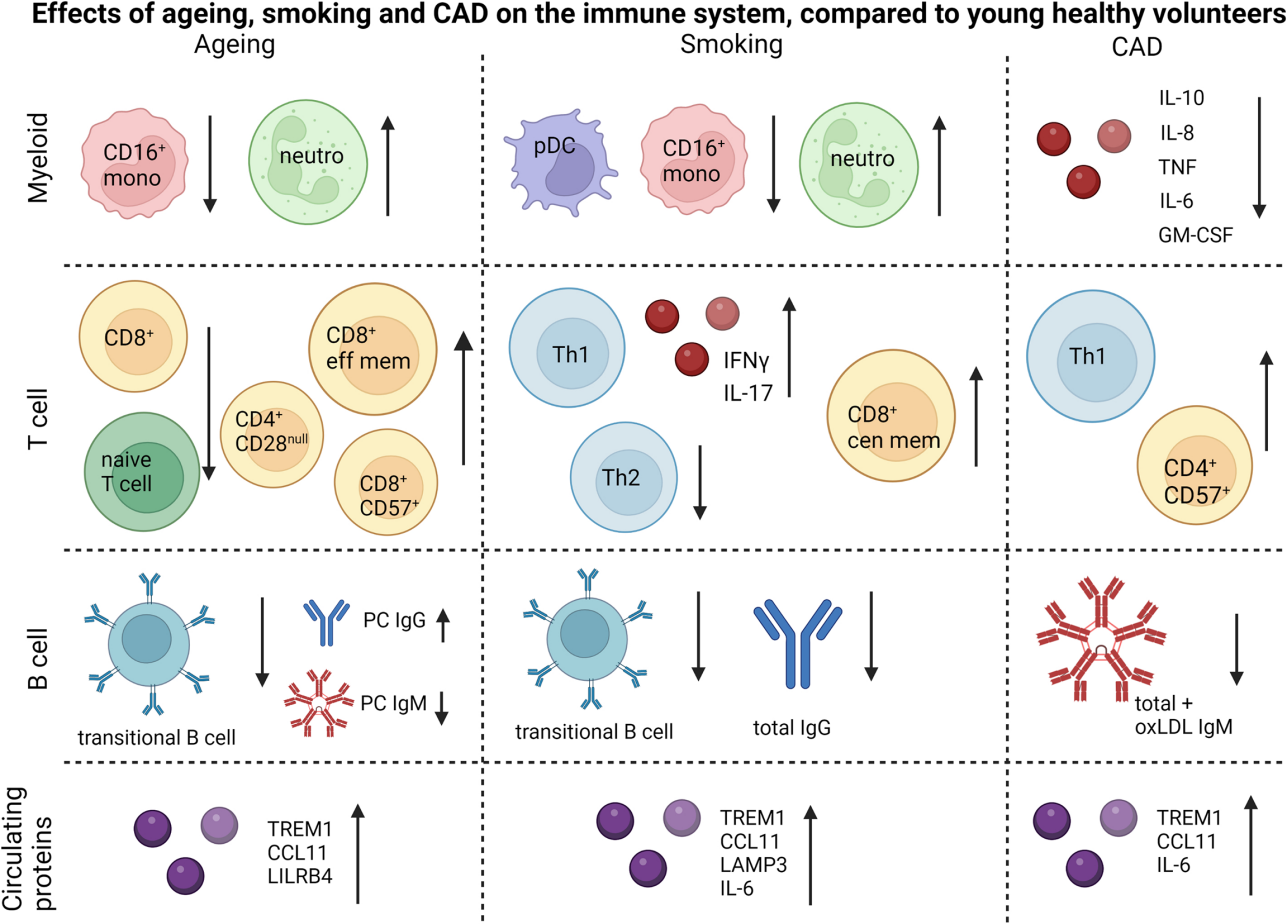
Summary of the main differences caused by ageing, smoking and CAD. Figure is created using biorender.com.

To summarize the study outcomes, we have visualized the main impact of aging, smoking and CAD on myeloid, T and B cell subsets/functionality, and on circulating immune markers (**Figure 8**). To conclude, significant effects of ageing and smoking on the immune system could be identified at relatively low group sizes of 11-30 subjects per group. Based on our data, both the aged and heavy smoking but otherwise healthy population, could be valuable for evaluation of the effects of future immunomodulatory drugs targeting atherosclerosis instead of patients with CAD, where the elderly healthy group corresponds most with the CAD patient group.

## Data availability statement

The original contributions presented in the study are included in the article/**Supplementary Material**. Further inquiries can be directed to the corresponding author.

## Ethics statement

The studies involving human participants were reviewed and approved by Stichting Beoordeling Ethiek Biomedisch Onderzoek, Assen, the Netherlands. The patients/participants provided their written informed consent to participate in this study.

## Author contributions

HG, PG, IB, JK, AF and MM designed the research. HG, VS, BH and CK acquired the data. HG, VS, CK and YY analyzed the data. HG, VS, AF and MM drafted the manuscript. All authors provided feedback on the research, analyses, and manuscript. All authors contributed to the article and approved the submitted version.

## Acknowledgments

The authors thank Maria Ozsvar Kozma for her assistance with serum immunoglobulin measurements. **Figure 8** was made using Biorender.com.

## Conflict of interest

The authors declare that the research was conducted in the absence of any commercial or financial relationships that could be construed as a potential conflict of interest.

## Publisher’s note

All claims expressed in this article are solely those of the authors and do not necessarily represent those of their affiliated organizations, or those of the publisher, the editors and the reviewers. Any product that may be evaluated in this article, or claim that may be made by its manufacturer, is not guaranteed or endorsed by the publisher.
