# Supplementary material for: Cardiovascular risk factors: The effects of ageing and smoking on the immune system, an observational clinical study
Source: Front Immunol. 2022 Sep 15;13:968815. doi: 10.3389/fimmu.2022.968815 (PMC9519851; doi:10.3389/fimmu.2022.968815)
Supplement: Supplementary file 1 [file DataSheet_1.docx]

Supplementary Material

Table S1: flow cytometry antibody list

| **Marker** | **Fluorochrome** | **Clone** | **Supplier** |
| --- | --- | --- | --- |
| CD1d | BV421 | 51.1 | Biolegend |
| CD3 | BV570 | UCHT1 | Biolegend |
| CD3 | BV650 | OKT3 | Biolegend |
| CD3 | AF700 | OKT3 | Biolegend |
| CD4 | BV650 | OKT4 | Biolegend |
| CD4 | BV510 | OKT4 | Biolegend |
| CD4 | BV605 | OKT4 | Biolegend |
| CD5 | AF700 | UCHT2 | Biolegend |
| CD8 | BV570 | RPA-T8 | Biolegend |
| CD8 | PE-Cy5 | HIT8a | Biolegend |
| CD11b | BV605 | ICRF44 | Biolegend |
| CD11c | Pacific blue | Bu15 | Biolegend |
| CD11c | PE-Cy5 | 3.9 | Biolegend |
| CD14 | Pacific blue | HCD14 | Biolegend |
| CD16 | PE-Cy7 | 3G8 | Biolegend |
| CD19 | BV605 | HIB19 | Biolegend |
| CD19 | AF700 | SJ25C1 | Biolegend |
| CD19 | FITC | HIB19 | Biolegend |
| CD123 | PE | 6H6 | Biolegend |
| CD127 | BV421 | A019D5 | Biolegend |
| CD138 | PE | MI15 | Biolegend |
| CD24 | BV605 | ML5 | Biolegend |
| CD24 | PE-Dazzle594 | ML5 | Biolegend |
| CD25 | VioBright515 | REA570 | Miltenyi |
| CD27 | AF700 | M-T271 | Biolegend |
| CD27 | FITC | M-T271 | Biolegend |
| CD27 | PECy7 | M-T271 | Biolegend |
| CD28 | PeCy7 | CD28.2 | Biolegend |
| CD38 | PE-Dazzle594 | HIT2 | Biolegend |
| CD38 | PE-Cy5 | HIT2 | Biolegend |
| CD43 | PE-Cy7 | CD43-10G7 | Biolegend |
| CD45ro | APC | UCHL1 | Biolegend |
| CD57 | PE | HNK-1 | Biolegend |
| CD66b | AF700 | G10F5 | Biolegend |
| CD70 | AF647 | 113-16 | Biolegend |
| CCR6 | BV650 | G034E3 | Biolegend |
| CCR7 | PE | G043H7 | Biolegend |
| CXCR3 | PE-Cy7 | G025H7 | Biolegend |
| HLA-DR | BV605 | L243 | Biolegend |
| IgD | FITC | IA6-2 | Biolegend |
| IgG | BV570 | M1310G05 | Biolegend |
| IgM | BV421 | MHM-88 | Biolegend |
| **intracellular markers** |  |  |  |
| IL-4 | PE | 8D4-8 | Biolegend |
| IL-17A | AF488 | BL168 | Biolegend |
| IFNy | PE-dazzle594 | 4S.B3 | Biolegend |
| IL-10 | APC | JES3-19F1 | Biolegend |
| IL-35/IL-27 | PE | B032F6 | Biolegend |

PE = Phycoerythrin, BV = Brilliant Violet, AF = Alexa Fluor, FITC = Fluorescein isothiocyanate, APC = allophycocyanin

Table S2: cytokine release levels after whole blood LPS stimulation

| **Cytokine** | **YH** | **EH** | **YS** | **HS** | **CAD** |
| --- | --- | --- | --- | --- | --- |
| TNF  (mean ± SD) | 7169  ± 4742 | 4138  ± 2764 | 4527  ± 2540 | 3950  ± 3822 | 4089  ± 2137 |
| GM-CSF  (mean ± SD) | 860.6  ± 476.8 | 656.2  ± 238.1 | 577.1  ± 244.4 | 497.9  ± 261.3 | 440.7  ± 266.4 |
| IL-8  (mean ± SD) | 7561  ± 4477 | 6126  ± 2704 | 6050  ± 2419 | 5991  ± 2660 | 4460  ± 1827 |
| IL-6  (mean ± SD) | 31821  ± 14073 | 24488  ± 10614 | 24641  ± 10729 | 24453  ± 11592 | 22664  ± 11740 |
| IL-10  (mean ± SD) | 379.3  ± 334.2 | 277.1  ± 247.7 | 341.3  ± 250.8 | 335.9  ± 185.4 | 175.3  ± 129.4 |





**Supplementary Figure 1: effect of ageing and smoking on myeloid cells**

Percentage of classical monocytes (CD14^+^CD16^-^), intermediate monocytes (CD14^+^CD16^+^) and myeloid dendritic cells (HLA-DR^+^CD14^-^CD11c^+^) in whole blood as assessed by flow cytometry (A). Whole blood was stimulated with 2 ng/ml LPS and IL-1β release was measured using LegendPlex (B). YH = young healthy, EH = elderly healthy, YS = young smoker, HS = heavy smoker, CAD = coronary artery disease patient. Open circles in the CAD group represent smokers. Statistics was performed using one-way ANOVA with Dunnett’s post hoc test, means were compared to the YH group.





**Supplementary Figure 2: effect of ageing and smoking on regulatory T cells**

Percentage of regulatory T cells (CD4^+^CD25^+^CD127^-^) in whole blood as assessed by flow cytometry. YH = young healthy, EH = elderly healthy, YS = young smoker, HS = heavy smoker, CAD = coronary artery disease patient. Open circles in the CAD group represent smokers. Statistics was performed using one-way ANOVA with Dunnett’s post hoc test, means were compared to the YH group.





**Supplementary Figure 3: effect of ageing and smoking on B cells**

Percentage of naïve B cells (CD27^-^IgD^+^), non-class-switched B cells (CD27^+^IgD^+^), class-switched B cells (CD27^+^IgD^-^IgM^-^) and B1 cells (CD27^+^CD43^+^) in whole blood as assessed by flow cytometry (A). PBMCs were stimulated with CpG class B for 24 hours, followed by a 4 hour stimulation with PMA and ionomycin in the presence of brefeldin A. Intracellular staining was performed to measure IL-10 and IL-35/IL-27 production in B cells (B). CD69 and CD86 expression was measured by flow cytometry on isolated B cells after 24 hours of stimulation with CpG class B (C). YH = young healthy, EH = elderly healthy, YS = young smoker, HS = heavy smoker, CAD = coronary artery disease patient. Open circles in the CAD group represent smokers. Statistics was performed using one-way ANOVA with Dunnett’s post hoc test, means were compared to the YH group.

**
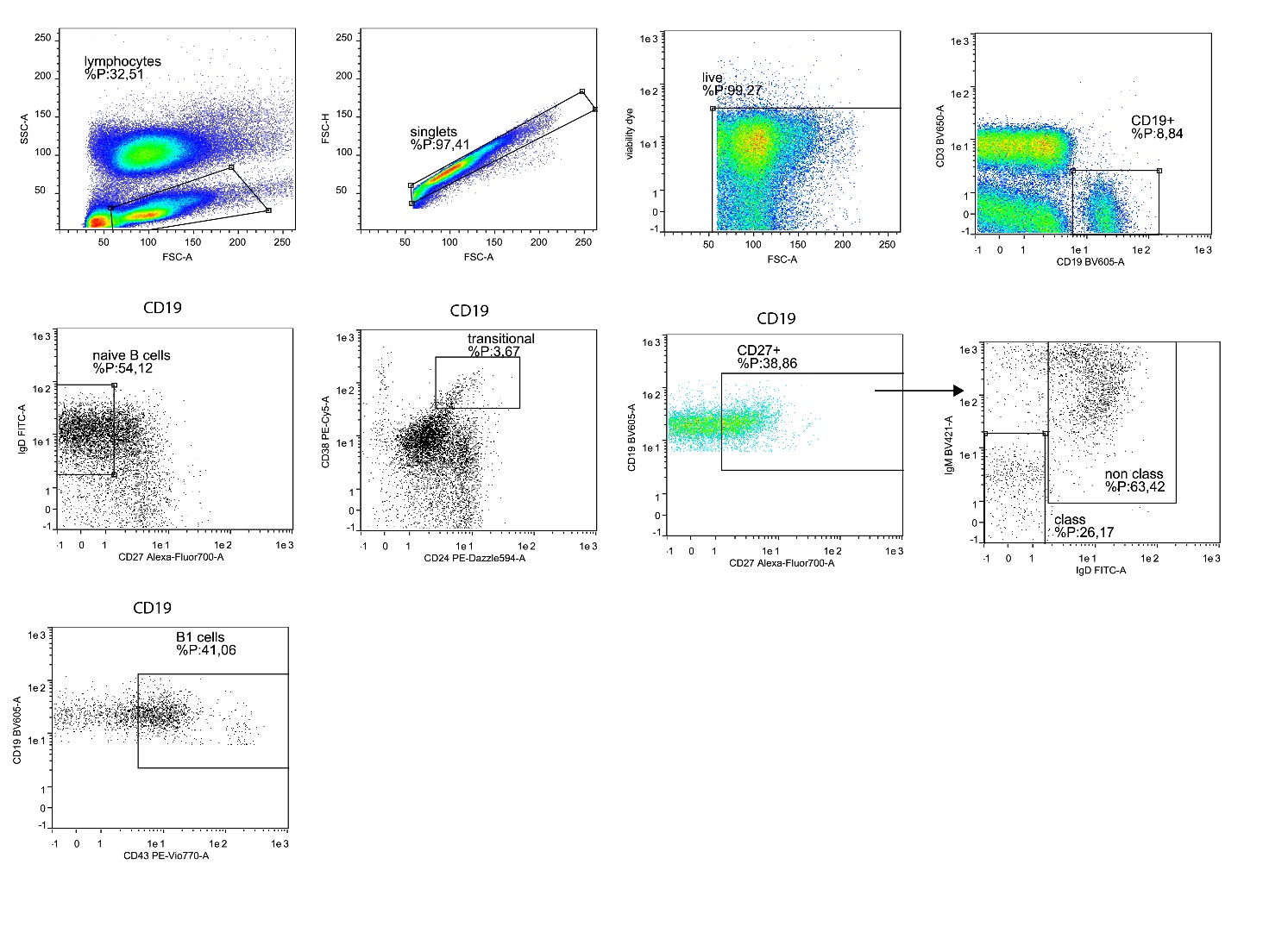
**

**Supplementary Figure 4: gating strategy B cells**

First, lymphocytes were gated in the FSC/SSC plot, followed by singlets and live cells. Within the CD19^+^ B cell population, naïve B cells were gated as IgD^+^ and CD27^-^. Transitional B cells were gated as CD38^+^CD24^+^. Within the CD27^+^ memory fraction, non-class-switched B cells were gated as IgM^+^IgD^+^, while class-switched B cells were gated as IgM^-^IgD^-^. Lastly, CD43^+^ B1 cells were gated in the CD19^+^ fraction.


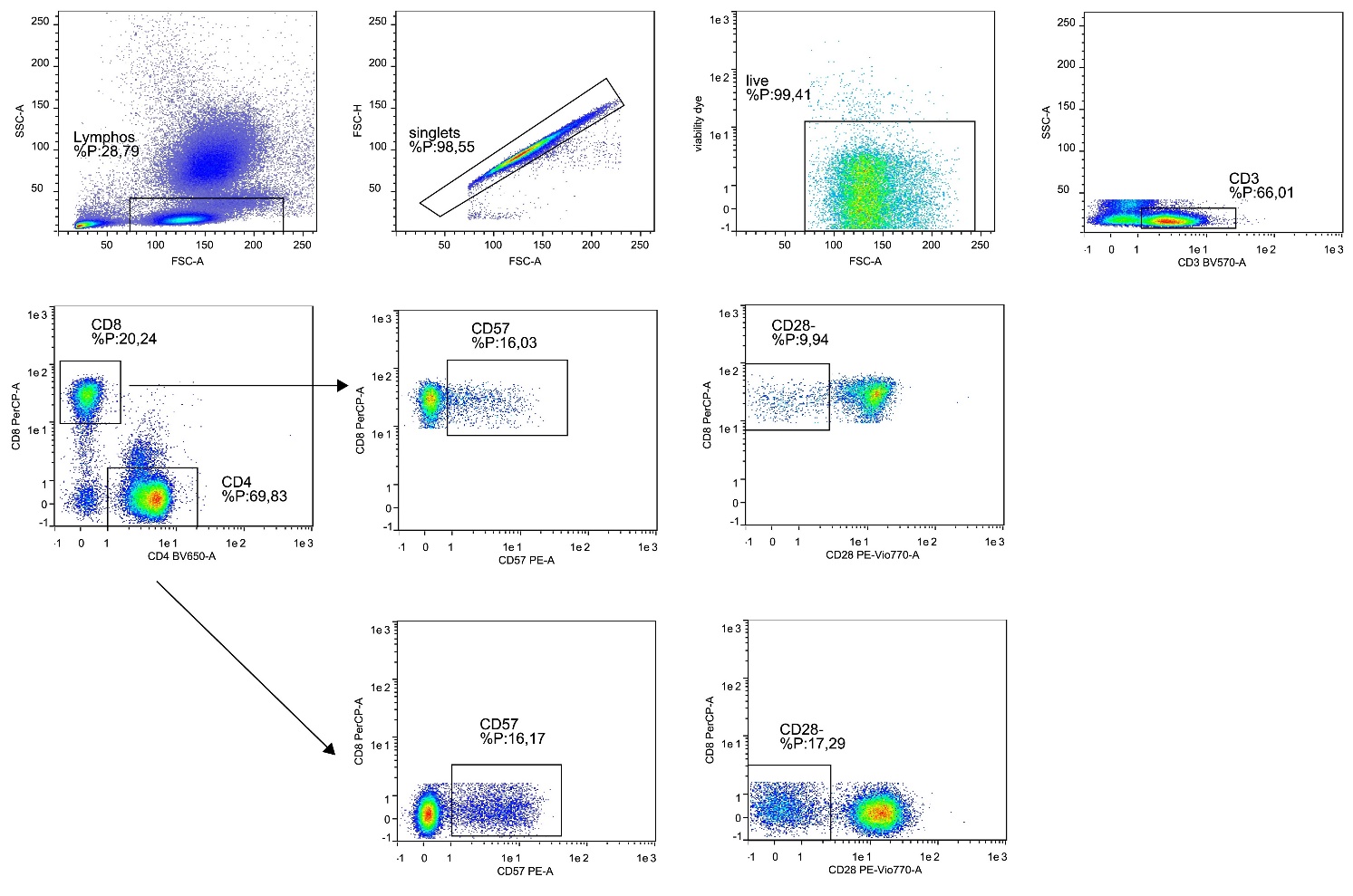


**Supplementary Figure 4: gating strategy senescent T cells**

First, lymphocytes were gated in the FSC/SSC plot, followed by singlets and live cells. Within the CD3^+^ T cell population, CD4^+^ and CD8^+^ T cells were gated. In both the CD4 and CD8 populations, CD57^+^ cells, and CD28^-^ cells were gated.


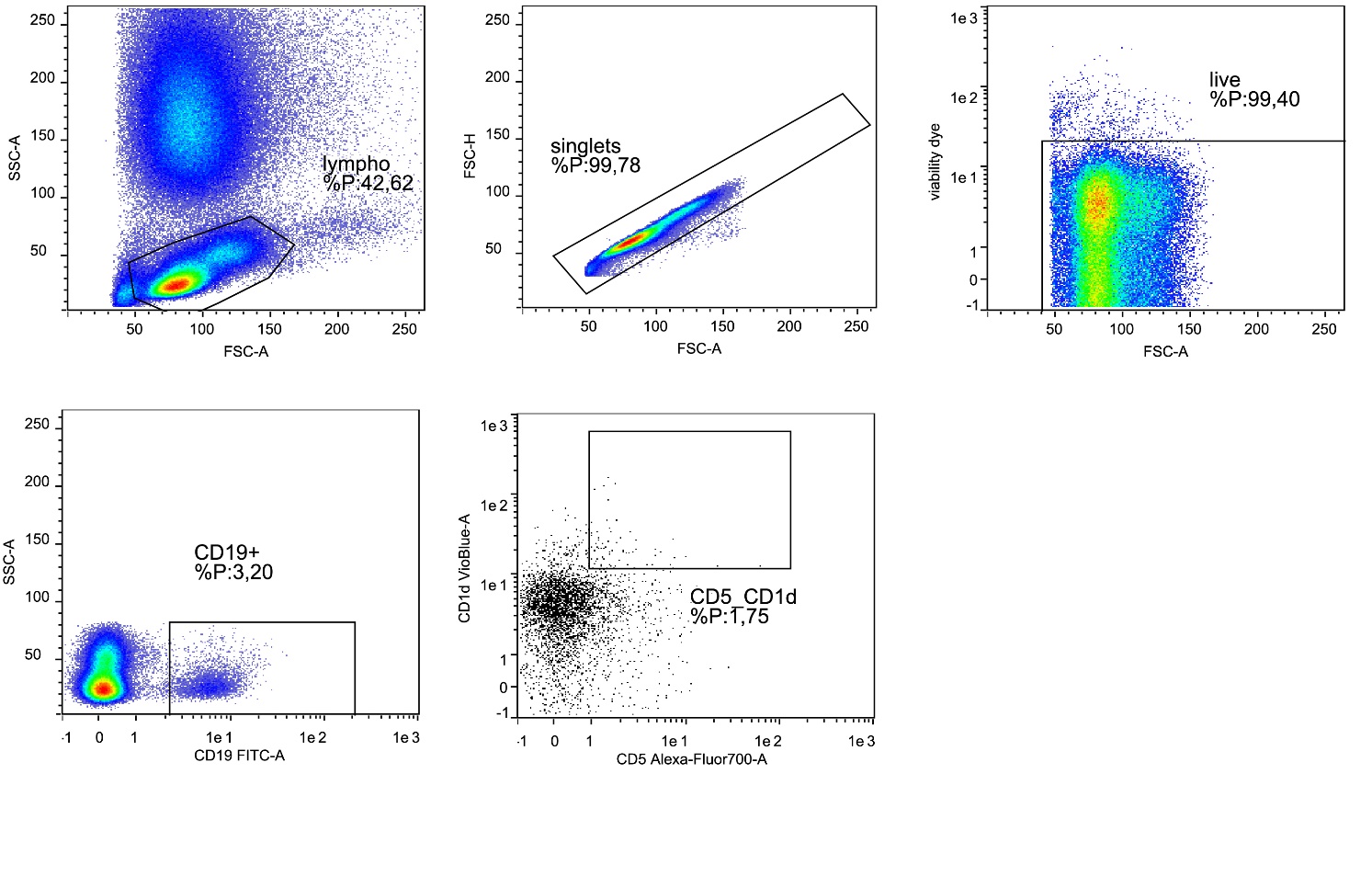


**Supplementary Figure 5: gating regulatory B cells**

First, lymphocytes were gated in the FSC/SSC plot, followed by singlets and live cells. Within the CD19+ B cell population, CD5^+^CD1d^hi^ cells were gated.

**
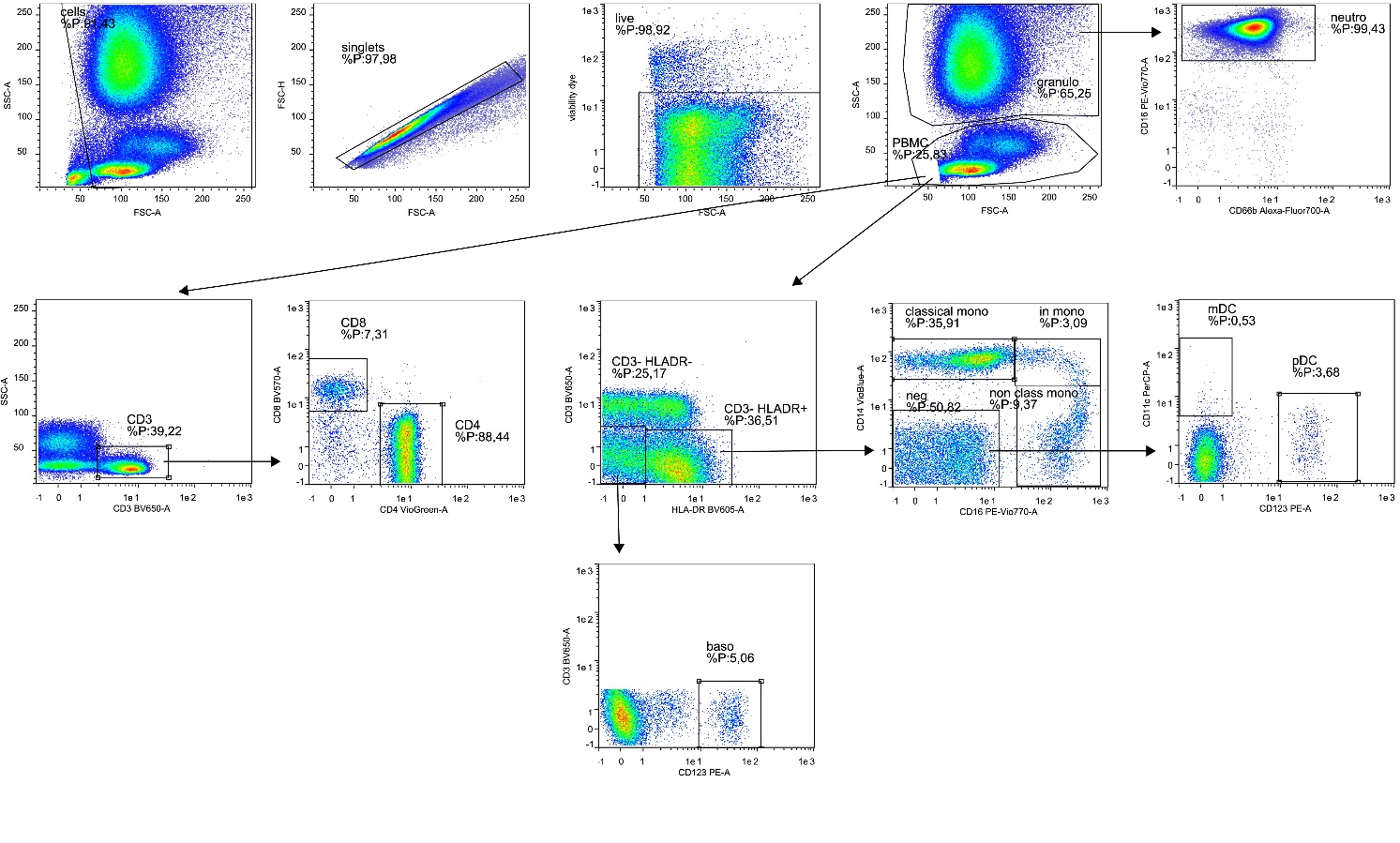
**

**Supplementary Figure 5: gating general immune cell subsets**

First, all cells were gated in the FSC/SSC plot, followed by singlets and live cells. Within the FSC/SSC plot, granulocytes and PBMCs were gated separately. Within the granulocyte gate, neutrophils were gated as CD16^+^CD66b^+^. Within the PBMC gate, CD3+ T cells were gated, followed by CD4^+^ and CD8^+^ T cells. Also within the PBMC gate, HLA-DR^+^ and HLA-DR^-^ cells were gated, both negative for CD3. Within the HLA-DR^-^ gate, basophils were gated as CD123^+^. Within the HLA-DR^+^ gate, monocytes were gated, CD14^+^ for classical monocytes, CD14^+^CD16^+^ for intermediate monocytes and CD16^+^ for non-classical monocytes. mDCs (CD11c^+^) and pDCs (CD123^+^) were gated from monocyte^-^ events.


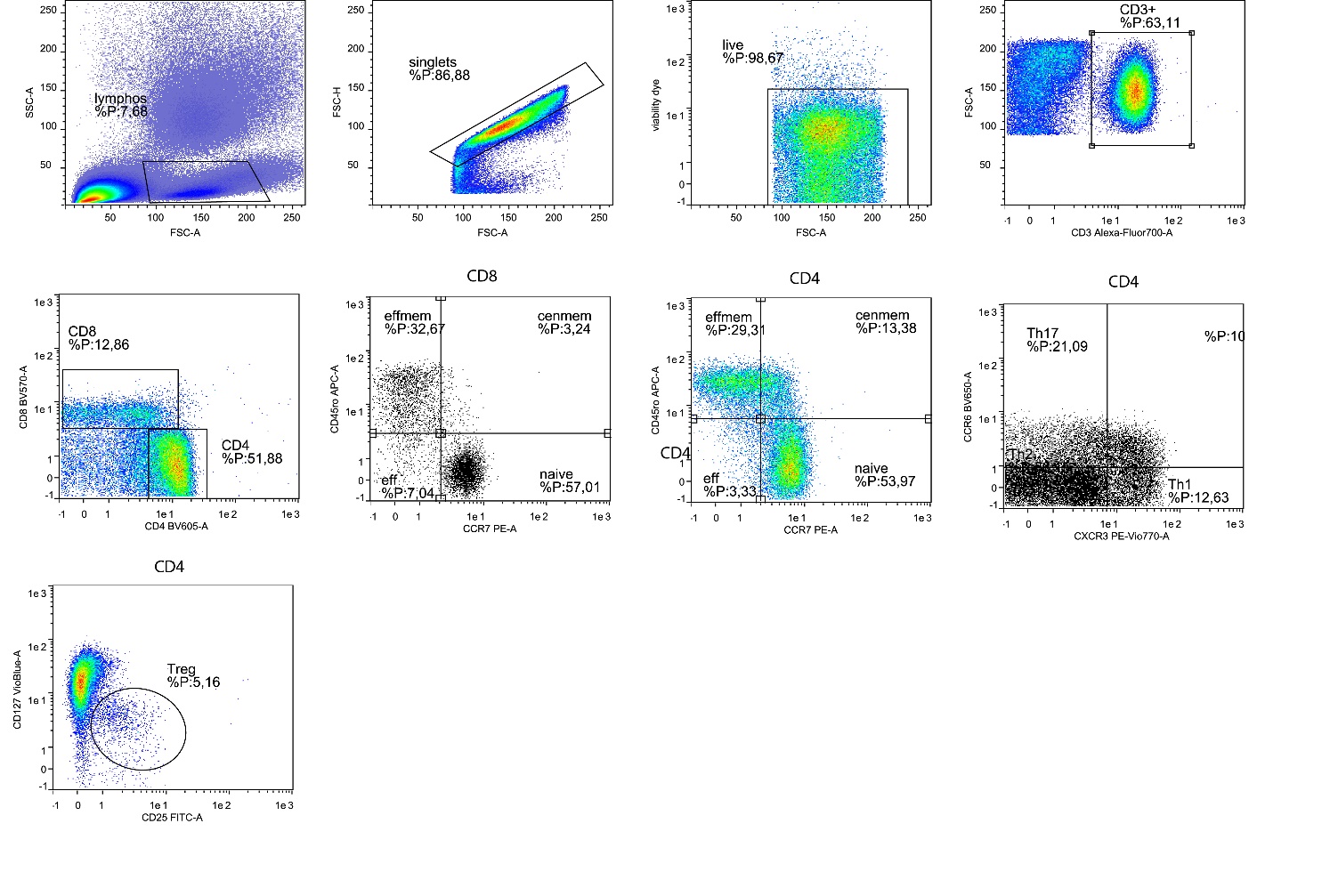


**Supplementary Figure 6: gating T cell subsets**

First, lymphocytes were gated in the FSC/SSC plot, followed by singlets and live cells. CD4 and CD8 T cells were gated from the CD3 T cell gate. Within the CD8 T cells, effector memory (CD45ro^+^CCR7^-^), central memory (CD45ro^+^CCR7^+^) and naïve (CCR7^+^CD45ro^-^) T cells were gated based on CCR7 and CD45ro expression. Memory subsets were also measured within the CD4^+^ population in the same way as for CD8. T helper subsets were assessed within the CD4+ population using CCR6 and CXCR3 antibodies, where Th17 cells are CCR6^+^ CXCR3^-^, Th1 cells CCR6^-^ CXCR3^+^ and Th2 cells CCR6^-^CXCR3^-^. Lastly, regulatory T cells were gated as CD25^+^CD127^-^ in the CD4 population.


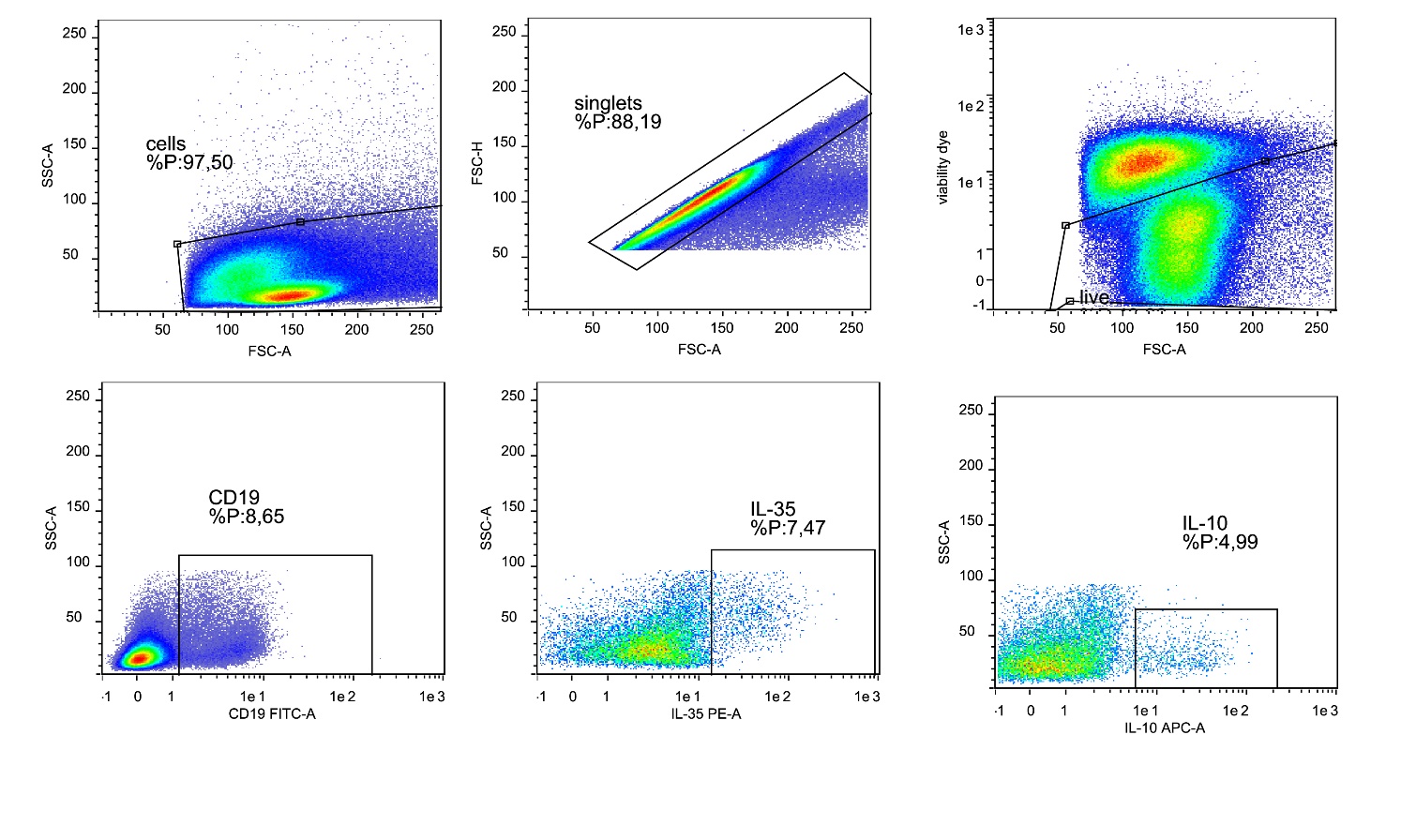


**Supplementary Figure 7: intracellular IL-10 and IL-35 B cell staining**

First, all cells were gated in the FSC/SSC plot, followed by singlets and live cells. Within the CD19^+^ B cell population, IL-35 and IL-10 gating was performed.


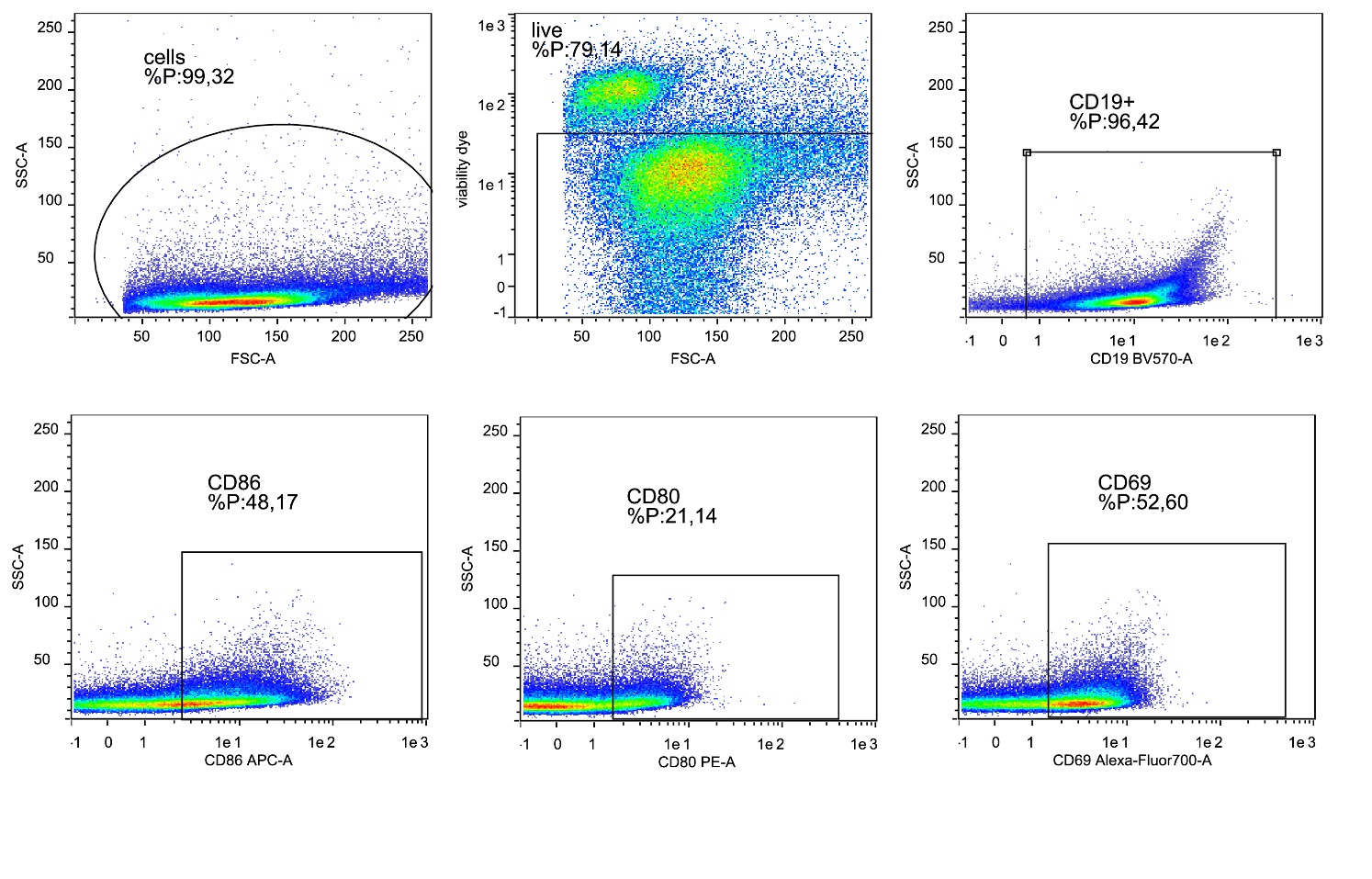


**Supplementary Figure 8: B cell activation marker staining**

First, all cells were gated in the FSC/SSC plot, followed by live cells. Within the CD19^+^ B cell population, activation markers CD86, CD80 and CD69 were gated.


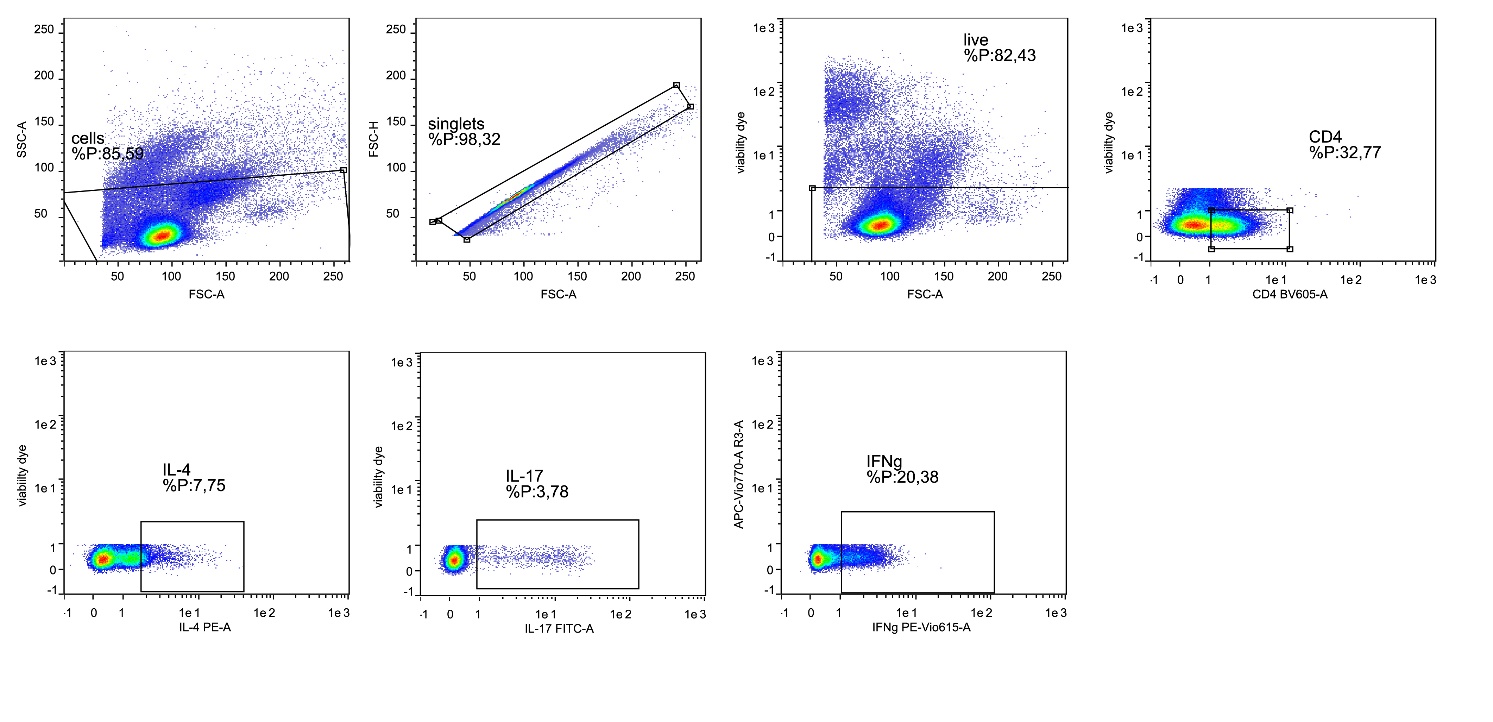


**Supplementary Figure 9: intracellular T cell staining**

First, all cells were gated in the FSC/SSC plot, followed by singlets and live cells. Within the CD4^+^ gate, IL-4, IL-17 and IFNγ staining was gated.
